# Association between Experiences in Life and Quality of Life among Lebanese University Students in a Collapsing Country: The Moderating Role of Religious Coping and Positivity

**DOI:** 10.3390/healthcare11010149

**Published:** 2023-01-03

**Authors:** Sara Moussa, Diana Malaeb, Muna Barakat, Toni Sawma, Sahar Obeid, Souheil Hallit

**Affiliations:** 1 Faculty of Medicine, University of Balamand, Koura 3843, Lebanon; 2 College of Pharmacy, Gulf Medical University, Ajman P.O. Box 4184, United Arab Emirates; 3 Department of Clinical Pharmacy and Therapeutics, School of Pharmacy, Applied Science Private University, Amman P.O. Box 166, Jordan; 4 Social and Education Sciences Department, School of Arts and Sciences, Lebanese American University, Jbeil 1401, Lebanon; 5 School of Medicine and Medical Sciences, Holy Spirit University of Kaslik, Jounieh P.O. Box 446, Lebanon; 6 Research Department, Psychiatric Hospital of the Cross, Jal Eddib P.O Box 60096, Lebanon

**Keywords:** quality of life, religious coping, experiences in life, positivity, university students, Lebanon

## Abstract

(1) Background: No goal can be more important than optimal individual quality of life (QOL); life experiences, religious coping, and positivity play pivotal roles in achieving this goal. The aim is to assess the correlation between positive/negative experiences in life and QOL, taking into consideration the moderating role of positivity and positive/negative religious coping among a sample of Lebanese university students. (2) Methods: This cross-sectional study was carried out between November and December 2021, and enrolled 333 university students, aged between 18 and 30 years, from various areas in Lebanon’s governorates. The 12-item Short Form Health Survey, the brief religious coping methods scale, and the Scale of Positive and Negative Experience were included in the questionnaire. Forward linear regressions were conducted to check for correlates associated with physical and mental QOL scores. All variables that showed a correlation or effect size > |0.24| were entered in the multivariable and moderation models as independent ones. Significance was set at a *p* < 0.05. (3) Results: A linear regression, taking the physical QOL score as the dependent variable, showed that living in rural areas compared to urban and more positivity were significantly associated with a better physical QOL, whereas more negative religious coping was significantly associated with a lower physical QOL. More positivity and more positive religious coping were significantly associated with a better mental QOL. The moderation analysis showed that in people with high positive experiences, those with higher negative religious coping had lower mental QOL. (4) Conclusions: In a society as deeply religious as Lebanon, it is crucial to organize awareness programs to help in discriminating between religious coping mechanisms. Across order to stop further detrimental effects on QOL, our findings may serve as a solid foundation for future studies of QOL with additional variables, in other groups and nations.

## 1. Introduction

No goal can be more important than optimal individual functioning and well-being. Over the past two decades, efforts to achieve this endpoint have changed and become known as “quality of life” research [1]. The World Health Organization (WHO) defines quality of life (QOL) as “an individual’s perception of their position in life in the context of the culture and value systems in which they live and in relation to their goals, expectations, standards, and concerns” [2]. Due to the subjective nature of an individual’s QOL, and the inherent biased characteristics associated with its measurement, its incorporation into scientific study has always been a challenge [3]. In terms of health, it has become a major concern for physicians that their decisions must consider the patient’s point of view and feelings towards the experiences he is about to go through, or in other terms, his QOL [4].

With time, researchers have reached a considerable agreement that QOL is multidimensional. It covers physical, material, social, and emotional well-being [5]. Life experiences, whether perceived as positive or negative, can disturb ongoing life and substantially affect one’s QOL [6]. A study on nursing students found that while extracurricular activities, campus life satisfaction, and task-related positive life events were statistically significant in predicting a better QOL, QOL was adversely correlated with task-related negative life events [7]. Indeed, stressful life events were generally related to poorer QOL [8]. Thus, previous literature supports the relation that exposure to different life experiences can affect QOL. This raises the recognition of the importance of timely psychological interventions in people with poor life events [9].

Religiosity, and especially the concept of religious coping, play a pivotal role in QOL [10]. In fact, the research orientation now prioritizes the influence of religion because it is viewed as a coping mechanism associated with favorable outcomes. Many people rely on their faith during difficult times [10]. However, depending on its adaptive mechanism, religious coping can be associated with a negative or positive outcome [11]. Pargament et al. [12] describe that when a strong religious basis is prominent such as “benevolent religious reappraisals, collaborative religious coping, seeking spiritual support, spiritual connection, religious purification, religious helping, and religious forgiving”; this is referred as positive religious coping. Negative religious coping, on the other hand, conveys a shaky faith in God, a menacing perspective of the world, and a need for significance. It has been demonstrated that those who are more spiritually attuned, more religious, and who visit church more frequently are more likely to have higher mental and emotional well-being levels [13]. In a sample of 143 depressed patients, intrinsic religiosity was found to be associated with more resilience and a better QOL [14]. Spiritual resources may contribute to greater QOL and health state, according to a cross-sectional study on hemodialysis patients [15]. Nowadays, more and more healthcare providers, notably midwives and nurses, leverage this connection and pay more attention to patients’ religious views in order to enhance their quality of life [16]. Therefore, one might draw the conclusion that religion, in particular religious coping, may be viewed as a significant element of and a contributing factor to QOL [17].

There have been decades of research on aspects of emotion such as positivity, as well as their relationship to concepts such as QOL, life satisfaction, and self-esteem. It has been postulated that a state of positivity whether in feelings of life satisfaction, coping, self-efficacy, has important implications for QOL assessment [18]. According to Fredrickson, having positive emotions helps people build a variety of enduring physical, intellectual, social, and psychological resources, which enables them to gradually improve their quality of life [19]. Indeed, positivity was linked to standards of ideal life satisfaction and well-being, so nations and people who highly valued positive emotions were more likely to show superior QOL [20]. Thus, prioritizing happiness in our daily lives, which shows up in how much we arrange our schedules to optimize our feelings of happiness, has been discovered to be connected with greater levels of QOL through feelings of well-being [21].

On one hand, previous research has found that life experiences are associated with QOL, while on the other hand, it found that religious coping and positivity are also associated with QOL. However, no studies have attempted to investigate the role of either religious coping or positivity in the association between life experiences and QOL among the Lebanese population. Also, there is an absence of studies examining each of the above variables in general within our population. Therefore, this study was conducted to investigate the relationship between religious coping, positivity, experiences in life, and QOL among a Lebanese adult sample. We hypothesize that religious coping and positivity might play a moderating role between experiences in life and QOL.

Lebanon is a developing country marred by several years of civil war and continuing political, social, and economic instability. The Beirut bombings deteriorated the situation in Lebanon and significantly impacted people’s quality of life, health, and living conditions [22]. Given the rapidly deteriorating situation in Lebanon, Lebanon’s residents’ quality of life is severely jeopardized. Lebanese people have been suffering from mental health issues before [23,24] and after the COVID-19 pandemic [25]. Researchers have agreed that mental health is greatly affected by social and economic instabilities, and Lebanon is no exception to that [26]. According to a study conducted on 2857 adults in Lebanon, almost half of the people had a history of experiencing quite horrific situations, which strongly influenced their mood, anxiety, and daily QOL [27]. Another study investigated the acute stress disorder (ASD) among different age ranges in Lebanon. Its results showed that young adults are the most affected (13.2%) compared with middle-aged adults (4.7%) and older respondents (4.3%) [28]. Finally, Lebanon is rolling from crisis to crisis, making long term plans for the youth a hard task especially after the Beirut Blast and the current COVID-19 pandemic. Limited attention has been given to the effect of the collapsing situation in Lebanon on the QOL of its young adults. According to a previous study conducted in Lebanon, youth’s positivity is strongly affected by age, gender, and residency, and the residential area will shape their life experiences and their ability to flourish and prosper [29]. The impact of Lebanon’s deteriorating status on its young people’ QOL has received little attention. In addition, little to no research has investigated the relationship between QOL and the different factors that affect it. The main objective of this study, the first of its kind in Lebanon, is to assess QOL among a sample of Lebanese university students and its association with experiences in life, taking into consideration the moderating role of religious coping and positivity during these times of economic crises and the COVID-19 dramatic illness on top of the daily stressors in the life of a Lebanese citizen.

## 2. Materials and Methods

### 2.1. Participants

This cross-sectional study was carried out between November and December 2021; it enrolled university students, aged between 18 and 30 years, who were recruited by convenience sampling from various areas in Lebanon’s governorates (Beirut, Mount Lebanon, South, North, and Beqaa). After the eligibility criteria had been determined and the consent form had been filled, 333 people who voluntarily took part in our study by accessing the survey’s online link after receiving it were included in the study. The research team started contacting people they know to enroll in this study; those people were then asked to forward the link to other friends and family members for participation, explaining the snowball technique. Those who refused to give consent were excluded. There was no benefit or compensation given to the participating individuals.

### 2.2. Minimal Sample Size Calculation

According to the G-power software, a minimum of 316 students was deemed necessary to have enough statistical power, based on a 5% risk of error, 80% power, f^2^ = 2.5%, and 10 factors to be entered in the multivariable analysis.

### 2.3. Questionnaire

This questionnaire compromised questions that covered different aspects; the first part included the socio-demographic of participants (age, gender, marital status, region of living), and the household crowding index (the number of rooms divided by the number of persons) [30]. Participants were asked to rate their financial burden using one question on a scale from 1 to 10, with 10 referring to overwhelming pressure. The second part included questions of well-known scales. This questionnaire needed to be translated because some of the scales were not validated. The Arabic version of the questionnaire needed 10–15 minutes to be completed. In order to ensure the accuracy of the translation, two healthcare specialists assisted the forward translation from English into Arabic that was carried out by a legal translator. Then, to rule out any discrepancy, the differences between the two English translations were then assessed, and a consensus was reached. Once ready, this questionnaire was transformed into Google Forms and then diffused among participants as a self-administered online questionnaire. The following scales were used in the second part of the questionnaire:

#### 2.3.1. The 12-Item Short Form Health Survey (SF-12)

The scores of the 12 items, which range from 0 for the lowest level of health to 100 for the highest level, are used to calculate the composite scores for physical (PCS) and mental (MCS) health [31]. The Arabic version of the SF-12 was used [32] (Cronbach’s alpha = 0.86).

#### 2.3.2. Positivity Scale

It consists of eight items, with scores ranging from 1 for strongly disagree to 5 for strongly agree. The greater the score, the more positive the outcome [33] (Cronbach’s alpha = 0.79).

#### 2.3.3. The Brief Religious Coping Methods Scale (Brief RCOPE Scale)

The author, Professor Kenneth Pargament, granted permission for this scale to be used and translated into Arabic. Positive and negative coping items are equally distributed across the 14 items that make up this scale [34] and scored from 0 to 7. Higher scores indicate higher religious coping (Cronbach’s alpha values were 0.90 for positive religious coping and 0.94 for negative religious coping).

#### 2.3.4. Scale of Positive and Negative Experience

The 12 items are divided between six positive feelings and six negative ones. Each item is scored from 0 to 5. The two scores can be combined to create a balance score in which the negative feelings score is subtracted from the positive feelings score, and the resultant difference score can vary from −24 (unhappiest possible) to 24 (highest affect balance possible) [35] (Cronbach’s alpha values were 0.88 for the positive subscale and 0.84 for the negative subscale).

### 2.4. Statistical Analysis

SPSS software version 23 was used to conduct data analysis. We had no missing data since all questions were required in the Google form. Cronbach’s alpha values were recorded for reliability analysis of all scales and subscales. The physical and mental QOL scores were normally distributed, with its skewness and kurtosis varying between −2 and +2 [36]. The Student’s t and ANOVA tests were used to compare two and three or more means respectively, whereas the Pearson correlation test was used to compare two continuous variables. Forward linear regressions were conducted to check for correlates associated with physical and mental QOL scores. The PROCESS MACRO 3.4 model 2 was used to assess the moderating effect of positivity and religious coping in the association between experiences in life and physical/mental QOL. All variables that showed a correlation or effect size > │0.24│ were entered in the multivariable and moderation models as independent ones to have a parsimonious model [37]. Significance was set at a *p* < 0.05.

## 3. Results

### 3.1. Sociodemographic and Other Characteristics of the Participants

A total of 333 students participated in this study; their mean age was 22.95 (SD: 4.79) years, with 65.8% females. Other characteristics are summarized in Table 1.

### 3.2. Bivariate Analysis

The outcomes of the bivariate analysis are displayed in Table 2 and Table 3. Both males and people who live in rural as opposed to urban regions had higher mean physical QOL scores. Additionally, males had a higher mean mental QOL score than females.

Our findings demonstrated that higher household crowding index, more negative religious coping, and more negative life experiences were significantly associated with less physical QOL, whereas higher financial burden, more positivity, and more positive life experiences were significantly associated with higher physical QOL. Additionally, lower mental QOL was significantly correlated with older age, higher household crowding index, more negative religious coping, and more negative life experiences, whereas higher mental QOL was correlated with higher positivity, more positive religious coping, and having more positive life experiences.

### 3.3. Multivariable Analysis

A linear regression, taking the physical QOL score as the dependent variable, showed that living in a rural area compared to urban (Beta = 2.91) and more positivity (Beta = 0.24) were significantly associated with a better physical QOL, whereas more negative religious coping (Beta = −0.30) was significantly associated with a lower physical QOL (Table 4).

More positivity (Beta = 0.30) and more positive religious coping (Beta = 0.48) were significantly associated with a better mental QOL (Table 5).

### 3.4. Moderation Analysis

**Figure 1:**
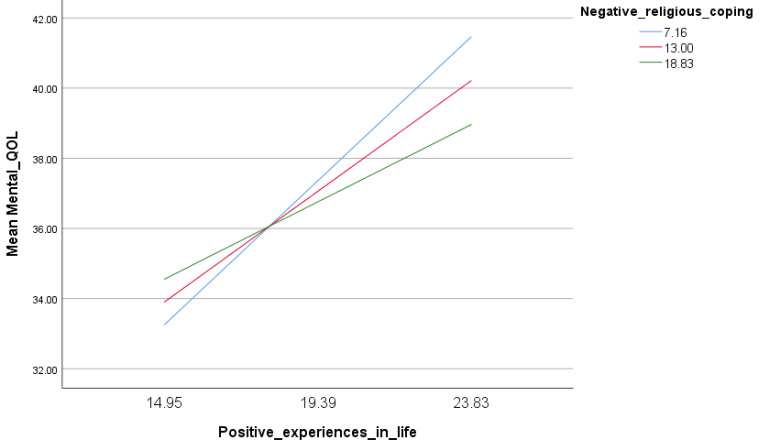
Association of the interaction of positive experiences in life by negative religious coping with mental quality of life.

A moderation analysis was conducted, taking positive/negative experiences in life as independent variables, positivity and positive/negative religious coping as concurrent moderators, and physical/mental QOL as dependent variables. The results showed that only the interaction negative religious coping by positive experiences in life was significantly associated with higher mental QOL (Beta = −0.04; t = −2.19; *p* = 0.029; 95% CI −0.07; −0.004); in people with high positive experiences in life, those with higher negative religious coping had lower mental QOL (Figure 1).

## 4. Discussion

This study confirmed earlier findings that religious coping had a considerable impact on mental QOL, with more positive religious coping being related with better mental QOL and more negative religious coping being associated with lower mental QOL [38,39,40,41,42,43,44,45]. Positive religious coping was directly associated to the greater QOL aspect of psychological health among people with schizophrenia, but poor religious coping and QOL were inversely related [38]. The same trend was observed in breast cancer [39], hemodialysis [40], HIV [41], and severely ill lung patients [42], who were at higher risks of a suboptimal QOL. The assessment of this association in nurses provided more support for this. The findings demonstrated that religious coping quality improvements brought about by regular training sessions would enhance nurses’ quality of life [43]. Furthermore, a cross sectional study showed that patients should be encouraged to use helpful religious resources, and that psycho-spiritual therapy should be tried to address religious conflicts that aim to improve QOL [44]. Finally, researchers describe it as ‘two sides of the same coin’; and if used positively, it may have positive outcomes on QOL [45].

Our results showed that positivity in life is correlated with a better mental QOL, corroborating previous studies [46,47,48,49,50,51,52]. In Italy, a total of 110 cancer patients were studied. The study concluded that positivity is a strength that accounts for an individuals’ QOL, even under severe diseases such as cancer [46]. Another study that described positivity as a potent predictor of health outcomes and QOL across a variety of functioning, self-esteem, and life satisfaction categories further supported this [47]. Similarly, when positivity was considered a protective factor for QOL, it was shown that it may attenuate the experience of distress at the time of disease diagnosis and may have a positive influence on QOL during and after treatment periods [48]. A quasi-experimental study conducted on students in Iran divided participants equally between experimental and control group. The experimental group, which was educated about positivity for eight sessions of 60 mins each, had an increased perception of competence and QOL in comparison with the control group [49]. This concept of positivity training was also tried on nurses. It was concluded that as long as nurses adopt a positive attitude towards their work and their work environment, it will improve their physical and mental health and QOL [50]. This was also true when applied to teachers. The use of positivity group training was shown to also increase the teachers’ QOL and resilience [51]. On a different note, Wissing et. al. wrote a chapter about the cultures of positivity where they argue that a good QOL and well-being is manifested in a culture of positivity [52].

Findings revealed that residents of rural areas had better physical QOL compared to those living in urban settings. The results related to this association are controversial; an Italian community survey showed that rural residence showed higher scores of QOL than urban residence [53]. The latter was also noticed in India, where older people who resided in urban areas reported much lower physical and psychological well-being levels than their rural counterparts [54]. On the other hand, an Australian survey showed that the overall QOL was lower in rural participants than for city dwellers, making rural living a significant negative predictor of QOL [55]. Another study conducted in India further proved this by showing that lower QOL in the domains of physical, psychological, environmental, and social relations was reported among individauls residing in the rural population [54]. This may be due to the major obstacles of the rural life such as the economic nature, unemployment, lack of services, and lower income compared to the city [56]. Given that, some researchers highlighted that immediate attention and interventions are necessary to improve rural QOL [57].

The moderation analysis in our study showed that in people with high positive experiences in life, those with higher negative religious coping had lower mental QOL. In general, people with high positive experiences in life tend to have an enhanced QOL [58]. Additionally, as discovered in other studies, poor overall QOL is linked to negative religious coping [59]. Not only that, a study involving 170 individuals with advanced cancer, which may be seen of as a negative life experience, discovered that worse overall QOL was associated with more frequently reported use of negative religious coping [60]. However, there were no research that looked at how negative religious coping affected the QOL of those with high levels of good experiences. Interestingly, the positive association between positive life experiences in life and QOL does not seem to be applicable in those with higher negative religious coping techniques. Thus, on one hand, we hypothesize that there may be a possible moderating effect of negative religious coping in negatively affecting the QOL of people with high positive life experiences. Additional research on life experiences and QOL might reveal more compelling moderators. On the other hand, since positive life experiences are associated with a better QOL and negative religious coping with a worse QOL; and since when these two were combined, the association yielded a better QOL, we also hypothesize that life experiences have a stronger effect on QOL than religious coping. Overall, the current findings will add to the existing body of knowledge and constitute a base for future research to build on.

The clinical implications of this study point to the need for experts to pay more attention to the variables affecting university students’ QOL. By enhancing their methods of religious coping and positivity levels, they would be aiming for a better QOL. Understanding these results will help implement awareness sessions to assist in differentiating between positive and negative religious coping methods, which is of upmost importance in a highly religious country such as Lebanon. Understanding the daily struggles that Lebanese young adults are up against will be the first step in developing preventative measures to face the exponential decrease in the youth’s QOL.

Our study has some limitations. First, due to its cross-sectional design, causation cannot be inferred. Second, some of the scales have not been validated, which required their translation to Arabic for the purpose of this study. With translation comes the risk of information bias because participants are more likely to underestimate or overestimate a question, which decreases the authenticity of the answer. This risk of information bias was further increased because the data has been obtained from a self-administered online questionnaire and because the quality of life, religious coping, and positivity are all subjective variables that differ from one person to another. Third, this study is subject to selection bias. In other words, the sample used was relatively small, not strictly between the age ranges that we were opting for, and we faced high refusal rates. Noteworthy, our study findings lack results extrapolation to the entire Lebanese young adult population. Thus, further studies with a larger sample size that aim to assess the associations noted in our study are to be conducted in the future. Finally, since some factors that may be associated with QOL were not evaluated in our study may introduce residual confounding bias.

## 5. Conclusions

Our findings indicate that positivity, positive religious coping, and living in rural areas exert an important positive influence on QOL, and identify groups (those who use negative religious coping methods and those living in urban areas) that are at greater risk for worse QOL. Unexpectedly, the interaction of negative religious coping by positive experiences in life was significantly associated with higher mental QOL, which is an important finding to build future research on. The study results are of upmost importance currently in Lebanon where the youth is experiencing severe levels of distress, in a country with minimal resources and opportunities. Lebanon is probably going to face a breakdown of mental health before one can notice, thus, further studies on a wider spectrum should be conducted to support the mental health field and help prevent further negative outcomes on QOL. Lastly, our findings will provide future investigations of QOL with possibly other variables, in different populations and in different countries.

## Figures and Tables

**Table 1:** Sociodemographic and other characteristics of the participants (*n* = 333).

Variable	*n* (%)
**Sex**	
Male	114 (34.2%)
Female	219 (65.8%)
**Marital status**	
Single	
Married	
**Region of living**	
Urban	210 (63.1%)
Rural	123 (36.9%)
	**Mean ± SD**
**Age (in years)**	22.95 ± 4.79
**Household crowding index**	1.09 ± 0.55
**Financial burden**	5.68 ± 2.66
**Physical quality of life**	44.77 ± 7.98
**Mental quality of life**	40.34 ± 8.95

**Table 2:** Bivariate analysis of the categorical variables associated with physical and mental quality of life (QOL).

Variable	Physical QOL			Mental QOL		
	Mean ± SD	*p*	Effect Size	Mean ± SD	*p*	Effect Size
**Sex**		**0.014**	0.009		**0.008**	0.310
Male	46.13 ± 6.39			42.14 ± 8.66		
Female	44.06 ± 8.62			39.40 ± 8.98		
**Marital status**		0.223	0.166		0.359	0.102
Single	45.04 ± 7.91			40.18 ± 9.69		
Married	43.69 ± 8.26			40.97 ± 4.49		
**Region of living**		**<0.001**	0.689		0.270	0.137
Urban	43.31 ± 8.52			40.72 ± 8.49		
Rural	48.17 ± 5.17			39.45 ± 9.94		

Numbers in bold indicate significant *p*-values.

**Table 3:** Bivariate analysis of the continuous variables associated with positivity.

Variable	Physical QOL		Mental QOL	
	r	*p*	r	*p*
Age	−0.106	0.053	−0.156	**0.004**
Household crowding index	−0.213	**<0.001**	−0.179	**0.001**
Financial burden	0.235	**<0.001**	−0.044	0.429
Positivity score	0.270	**<0.001**	0.296	**<0.001**
Positive religious coping	−0.170	**0.002**	0.415	**<0.001**
Negative religious coping	−0.305	**<0.001**	−0.175	**0.001**
Positive experiences in life	0.266	**<0.001**	0.552	**<0.001**
Negative experiences in life	−0.182	**0.001**	−0.139	**0.011**

Numbers in bold indicate significant *p*-values; r = Pearson correlation coefficient.

**Table 4:** Stepwise linear regression taking the physical QOL score as the dependent variable.

	Beta	β	*p*	95% CI
Negative religious coping	−0.30	−0.22	**<0.001**	−0.45; −0.16
Region of living (rural vs. urban *)	2.91	0.17	**0.003**	1.02; 4.79
Positivity	0.24	0.15	**0.006**	0.07; 0.41
Nagelkerke R^2^ = 15.6%.				

* Reference group; Beta = unstandardized beta; β = standardized beta; CI = Confidence interval; numbers in bold indicate significant *p*-values.

**Table 5:** Stepwise linear regression taking the mental QOL score as the dependent variable.

	Beta	β	*p*	95% CI
Sex (females vs. males *)	−1.49	−0.08	0.113	−3.34; 0.36
Positivity	0.30	0.17	**0.001**	0.12; 0.48
Positive experiences in life	0.08	0.06	0.373	−0.10; 0.26
Positive religious coping	0.48	0.32	**<0.001**	0.29; 0.67
Nagelkerke R^2^ = 21.1%.				

* Reference group; Beta = unstandardized beta; β = standardized beta; CI = Confidence interval; numbers in bold indicate significant *p*-values.

## Data Availability

The datasets generated and/or analysed during the current study are not publicly available due to the authors do not have the right to share any data information as per their institutions’ policies but are available from the corresponding author on reasonable request.
